# Dislocation of the Thumb

**Published:** 1841-10

**Authors:** 


*Dislocation of the Thumb.*—At the last meeting of the Hunterian Society on the 9th of June, Mr. Adams directed the attention of the members to a novel proceeding which he had successfully employed at the London Hospital in the reduction of a dislocation backwards of the first phalanx of the thumb. In this case much extension in the ordinary manner had been employed, but without relief. The method consisted in drawing backwards, or extending, as far as possible, the thumb, so as to incline the back of the first and second phalanx on the back of the metacarpal bone; by this the proximal end of the first bone was more closely approximated to the distal end of the metacarpal bone. The thumb was then gradually brought forwards over the end of the metacarpal bone, at the same time that the end of the first phalanx was firmly held in its position. By this means the thumb itself becomes converted into a considerable lever, the fulcrum of which is the proximal end of the first phalanx: the reduction was accomplished with great ease.—*Ibid.*

